# Determinants of sexual activity and its relation to cervical cancer risk among South African Women

**DOI:** 10.1186/1471-2458-7-341

**Published:** 2007-11-27

**Authors:** Diane Cooper, Margaret Hoffman, Henri Carrara, Lynn Rosenberg, Judy Kelly, Ilse Stander, Lynnette Denny, Anna-Lise Williamson, Samuel Shapiro

**Affiliations:** 1 Women's Health Research Unit, School of Public Health and Family Medicine, Faculty of Health Sciences, University of Cape Town, Anzio Road, Observatory, 7925, Cape Town, South Africa; 2 Slone Epidemiology Unit, Boston University, Boston, USA; 3 Medical Research Council of South Africa, Tygerberg, South Africa; 4 Department of Obstetrics and Gynaecology, Faculty of Health Sciences, University of Cape Town, Anzio Road, Observatory, 7925, Cape Town, South Africa; 5 Institute of Infectious Disease and Molecular Medicine, Faculty of Health Sciences, University of Cape Town, Anzio Road, Observatory, 7925, Cape Town, South Africa; 6 National Health Laboratory Service, South Africa

## Abstract

**Background:**

Invasive cervical cancer is the commonest cause of cancer morbidity and mortality in South African women. This study provides information on adult women's sexual activity and cervical cancer risk in South Africa.

**Methods:**

The data were derived from a case-control study of hormonal contraceptives and cervical cancer risk. Information on age of sexual debut and number of lifetime sexual partners was collected from 524 incident cases and 1541 hospital controls. Prevalence ratios and adjusted prevalence ratios were utilised to estimate risk in exposures considered common. Crude and adjusted relative risks were estimated where the outcome was uncommon, using multiple logistic regression analysis.

**Results:**

The median age of sexual debut and number of sexual partners was 17 years and 2 respectively. Early sexual debut was associated with lower education, increased number of life time partners and alcohol use. Having a greater number of sexual partners was associated with younger sexual debut, being black, single, higher educational levels and alcohol use. The adjusted odds ratio for sexual debut < 16 years and ≥ 4 life-time sexual partners and cervical cancer risk were 1.6 (95% CI 1.2 – 2.2) and 1.7 (95% CI 1.2 – 2.2), respectively.

**Conclusion:**

Lower socio-economic status, alcohol intake, and being single or black, appear to be determinants of increased sexual activity in South African women. Education had an ambiguous effect. As expected, cervical cancer risk is associated with increased sexual activity. Initiatives to encourage later commencement of sex, and limiting the number of sexual partners would have a favourable impact on risk of cancer of the cervix and other sexually transmitted infections

## Background

In developing countries invasive cancer of the cervix is the commonest cause of cancer morbidity and mortality among women [1,2]. The South African age-standardized incidence is estimated to be 30/100,000 per year [2]. High-risk human papillomavirus (HPV) infection is a necessary cause[3]. HPV is sexually transmitted with a strong association existing between sexual activity and cervical cancer risk [4,5]. Only a small proportion of HPV infected women go on to develop squamous intraepithelial lesions or invasive cancer. Hence there must be co-factors to HPV infection that lead to the development of cervical cancer. There is scant information on sexual activity in adult South African women, despite high persistent levels of high risk HPV among older women [6]. In this paper patterns and determinants of sexual activity among adult South African women and their relationship to cancer of the cervix are described.

## Methods

Data were derived from a case-control study conducted between January 1998 and September 2001 among women aged < 60 years, resident for at least six months within 150 km of Cape Town in the Western Cape, South Africa. The study's main purpose was to assess the relationship between hormonal contraception and cervical cancer risk and details of the methods have been described elsewhere [7]. Written informed consent was obtained from participants and data was kept confidential. Ethical approval was obtained from the Research and Ethics Committee at the University of Cape Town and the Institutional Review Board at Boston University.

Women developing cervical cancer and using public health facilities were all treated at two tertiary care hospitals, in Cape Town. Incident cases were women with clinically evident and histologically confirmed invasive cervical epithelial cancer (stages 1b-IVb), diagnosed ≤ 6 months previously, with no previous history of any malignancy, presenting at the oncology clinics of these hospitals. Controls were treated and recruited in nongynecologic, nonobstetric wards in local hospitals, accounting for the wider range of hospitals for controls. Cases and controls were series-matched in a ratio of 1:3 on decade of age, race and geographic residence (urban or rural areas in same catchment geographical areas). Controls were selected to be representative of the same population from which the cases were drawn and were similar in a range of socio-demographic characteristics (see Table 1). Eligible controls were women with primary diagnoses such as trauma or acute infections, that have not been linked in previous studies, either positively or negatively to cervical cancer risk, to contraceptive use (e.g. trauma, appendicitis, disc disease and other major orthopaedic conditions); selective admissions for major surgery for conditions such as inguinal hernia or to sexual practices: women with conditions such as ischemic heart disease or venous thromboembolism, or gynecologic disorders, were not eligible.

There were 535 potential cases and 1668 potential controls. Two cases (0.4%) and 107 controls (6.4%) refused participation: 38 of the latter were recently screened for cervical abnormalities and refused a repeat test after a short interval. Seven controls had stage 1A cervical cancer and were excluded from the study after referral for treatment. Permission was obtained from controls and endocervical scrapings taken for HPV testing for current infection with high risk HPV at the same time. These were assayed for HPV infection using the Hybrid Capture II test for detection of high risk HPV types [7]. HPV specimens were not taken from cases as many were deemed to be too sick at the time (a large proportion were stages 3 and 4 cancer) to engage in invasive specimen collection [7]. The high risk HPV status of controls only was therefore verifiable. White women were initially included in the study, but as only nine white cases and 14 controls were enrolled in the first year, the study was confined to coloured and black women. After exclusions, there were 524 cases and 1541 controls. The mean age of cases and controls was 45 years and 44 years, respectively; 75% of the women were coloured.

Trained nurses administered a standardized questionnaire in a face-to-face interview in the subject's first language. Information was collected on a wide range of variables including demographic data; life-time contraceptive (including history of condom use as a marker of safer sex) and reproductive history (including parity and past history of own and partners' sexually transmitted infections); cervical dysplasia history; PAP smear history; weight; height; smoking (ever smoked; number of years smoked, if ceased smoking, time since smoked and number of cigarettes if current smoker); alcohol consumption; and sexual activity history including recognized key indicators of sexual behaviour [8] age of sexual debut (penetrative sexual intercourse) and number of lifetime sexual partners.

**Table 1:** comparison of socio-demographic background of cases and controls

Characteristics	Cases (N = 524)	Controls (N = 1541)
*Mean age (and standard deviation)*	45 years (8.5)	44 years (8.7)
	N (%)	N (%)
*Residence*		
Rural	221 (42)	712 (46)
Urban	303 (58)	829 (54)
*Marital status*		
*Race*		
Black	133 (25)	386 (25)
Coloured	391 (75)	1155 (75)
*Years of education*		
≤ 4 years	129 (25)	431 (28)
5–9 years	319 (61)	857 (56)
≥ 10 years	76 (15)	253 (16)
*Marital status*		
Single	128 (25)	346 (23)
Divorced/separated	80 (15)	229 (15)
Widowed	76 (15)	180 (12)
Married	239 (46)	786 (51)

Age at first sex was an ordinal rather than a continuous variable collected three categories (< 16 years; 16–19 years and ≥ 20 years). This split was practically necessary to ensure adequate numbers by variable categories for analysis. Descriptive associations between the two main sexual activity indices that we created (age at first sex < 16 years and 3+ sexual partners) are presented as odd ratios derived from logistic regression analyses in Tables 2 and 3. Case-control methods were employed for data presented in Tables 4 and 5. Prevalence ratios and adjusted prevalence ratios were utilised to estimate risk in Tables 2 and 3 as some of these exposures were considered common (> 10%). These were estimated using an experimental Proc TPHREG in SAS 9.1.3. In the instance where the outcome is uncommon, as is the case in Tables 4 and 5 for cervical cancer risk, (estimated incidence of 30/100,000) crude and adjusted odd ratios were most likely to be a good estimate of relative risk. We assessed the association of potential cervical cancer risk factors with age at sexual debut using unconditional multiple logistic regression to adjust for confounding and estimated odds ratios for first sex at age < 16 years relative to first sex at a later age. Similar analyses assessed associations between potential risk factors and ≥ 3 lifetime sexual partners. Logistic regression assessed determinants of sexual activity to risk of cervical cancer. We controlled for age, race, marital status, progestogen only contraceptives (IPC) use, combined estrogen/progestogen contraceptive use (COC), years of formal education, life-time smoking and alcohol consumption and life-time pap smear history. Crude and adjusted relative risks (odd ratios) were estimated using multiple logistic regression analysis. The crude and adjusted relative risk estimates were similar and both are presented.

**Table 2:** Socio-demographic factors associated with earlier sexual debut

Factor	Total*	Age at sexual debut < 16 yrs (N = 357)	Crude PR (95% CI)	Adjusted PR ** (95% CI)
*Age*	N	N (%)		
< 30	97	23 (24)	0.9 (0.6 – 1.4)	1.5 (0.9 – 2.4)
30–39	373	81 (22)	0.8 (0.6 – 1.1)	1.0 (0.7 – 1.4)
40–49	605	43 (24)	0.9 (0.7 – 1.2)	1.0 (0.7 – 1.3)
50–59	425	110 (26)	[1.0] ***	[1.0]*
*Marital status*				
Single	337	72 (21)	0.9 (0.7 – 1.2)	0.9 (0.7 – 1.2)
Divorced	221	49 (22)	0.9 (0.7 – 1.3)	1.0 (0.7 – 1.4)
Widowed	171	49 (29)	1.2 (0.9 – 1.6)	1.2 (0.8 – 1.6)
Married	771	187 (24)	[1.0]*	[1.0]*
*Race*				
Black	351	89 (25)	1.1 (0.9 – 1.4)	1.2 (0.9 – 1.6)
Coloured	1149	268 (23)	[1.0] ***	[1.0]***
*Area of residence*				
Urban	801	172 (22)	[1.0] ***	[1.0]***
Rural	699	185 (26)	1.2 (1.0 – 1.5)	1.2 (0.9 – 1.5)
*Years formal education*				
< 4	412	152 (37)	3.8 (2.5 – 5.9)	4.1 (2.6 – 6.6)
5–9	837	181 (22)	2.3 (1.5 – 3.5)	2.4 (1.6 – 3.8)
≥ 10	251	24 (10)	[1.0]*	[1.0]***
*Ever use of IPCs*^+^				
Yes	1170	275 (24)	0.9 (0.7 – 1.2)	1.0 (0.8 – 1.3)
Never	330	82 (25)	[1.0] ***	[1.0]***
*Ever COC use*^++^				
Yes	592	120 (20)	0.8 (0.6 – 1.0)	0.9 (0.7 – 1.1)
Never	908	237 (26)	[1.0]*	[1.0]***
*Ever had PAP*				
Yes	1113	249 (22)	0.8 (0.6 – 1.0)	1.0 (0.8 – 1.3)
Never	386	108 (28)	[1.0] ***	[1.0]***
*Smoking status*				
Never smoked	602	131 (22)	[1.0] ***	[1.0]***
Ex-smoker	175	41 (23)	1.1 (0.8 – 1.5)	0.9 (0.6 – 1.3)
Current smoker	723	185 (26)	1.2 (0.9 – 1.5)	0.9 (0.7 – 1.2)
*Alcohol use*				
Never	721	134 (19)	[1.0] ***	[1.0]***
Past use	271	66 (24)	1.3 (1.0 – 1.8)	1.1 (0.8 – 1.5)
Current use	508	157 (31)	1.7 (1.3 – 2.1)	1.4 (1.1 – 1.8)
*Number sexual partners*				
1	410	65 (16)	[1.0]*	[1.0]***
2	510	122 (24)	1.5 (1.1 – 2.0)	1.3 (1.0 – 1.7)
3	301	78 (26)	1.6 (1.1 – 2.2)	1.4 (1.0 – 1.9)
≥ 4	275	89 (32)	1.9 (1.4 – 2.7)	1.9 (1.3 – 2.7)
*High risk HPV*				
*Positive*	244	63 (26)	1.1 (0.8–1.5)	1.0 (0.7–1.3)
*Negative*	1255	294 (23)	[1.0]*	[1.0]*

* Totals vary because of unknown values

** Adjustment: for all other factors in the table ; *** Reference category

+ Injectable Progesterone only contraceptives ++ Combined Hormonal Oral Contraceptives

**Table 3:** Socio-demographic factors associated with increased number of sexual partners

Factor	Total*	Number sexual partners ≥ 3 (N = 595)	Crude PR (95% CI)	Adjusted PR (95% CI)**
	N	N (%)		
*Age*				
< 30	99	45 (45)	1.3 (1.0 – 1.9)	0.9 (0.6 – 1.3)
30–39	380	162 (43)	1.3 (1.0 – 1.6)	1.0 (0.8 – 1.3)
40–49	619	240 (39)	1.1 (0.9 – 1.4)	1.0 (0.8 – 1.3)
50–59	435	148 (34)	[1.0]***	[1.0]***
*Marital status*				
Single	341	173 (51)	1.5 (1.3 – 1.9)	1.3 (1.1 – 1.6)
Divorced	227	98 (43)	1.3 (1.0 – 1.6)	1.1 (0.9 – 1.4)
Widowed	180	65 (36)	1.1 (0.8 – 1.4)	1.1 (0.8 – 1.4)
Married	784	259 (33)	[1.0]***	[1.0]***
*Race*				
Black	381	240 (63)	2.0 (1.7 – 2.4)	1.9 (1.5 – 2.3)
Coloured	1 152	355 (31)	[1.0]***	[1.0]***
*Area of Residence*				
Urban	823	356 (43)	[1.0]***	[1.0]***
Rural	710	239 (34)	0.8 (0.7 – 0.9)	0.9 (0.8 – 1.1)
*Years of formal education*				
≤ 4	427	140 (33)	1.0***	[1.0]***
5–9	854	347 (41)	1.2(1.0–1.5)	1.3 (1.0–1.5)
≥ 10	252	108 (43)	1.3 (1.0–1.7)	1.3 (1.0–1.7)
*Ever IPC use*^+^				
Yes	1195	478 (40)	1.2 (0.9 – 1.4)	1.0 (0.8 – 1.3)
Never	338	117 (35)	[1.0]***	[1.0]***
*Ever COC use*^++^				
Yes	602	223 (37)	0.9 (0.8 – 1.1)	1.0 (0.8 – 1.1)
Never	931	372 (40)	[1.0]***	[1.0]*
*Ever had PAP*				
Yes	1119	402 (36)	0.8 (0.7 – 0.9)	1.0 (0.8 – 1.2)
Never	413	192 (46)	[1.0]***	[1.0]***
*Smoking status*				
Never smoked	626	268 (43)	[1.0]***	[1.0]***
Ex-smoker	177	60 (34)	0.8 (0.6 – 1.1)	0.9 (0.7 – 1.2)
Current smoker	730	267 (37)	0.9 (0.7 – 1.0)	1.0 (0.8 – 1.2)
*Alcohol use*				
Never	739	226 (31)	[1.0]***	[1.0]***
Current use	280	112 (40)	1.3 (1.0 – 1.6)	1.6 (1.2 – 2.0)
Past use	514	257 (50)	1.6 (1.4 – 1.0)	1.7 (1.4 – 2.1)
*Age at first sex*				
< 16	354	167 (47)	2.7 (2.0 – 3.8)	2.4 (1.8 – 3.4)
16–19	837	357 (43)	2.5 (1.9 – 3.3)	2.1 (1.6 – 2.9)
≥ 20	305	52 (17)	[1.0]***	[1.0]***
*High risk HPV*				
*Positive*	253	117 (47)	1.2 (1.0–1.5)	1.1 (0.9–1,3)
*Negative*	1279	477 (37)	[1.0]***	[1.0]***

* Totals vary because of unknown values ** Adjustment: for all other factors in the table.

*** Reference category + Injectable Progesterone only contracaptives ++ Combined Hormonal Oral Contraceptives

**Table 4:** Cervical cancer risk in relation to age of sexual debut

	Age of sexual debut	Cases (N = 524)	Controls (N = 1541)	Crude OR CI (95%)	Adjusted OR* CI (95%)
		N (%)	N (%)		
All women**					
	≥ 20 years	76 (15)	305 (20)	[1.0]***	[1.0]***
	16–19 years	266 (51)	838 (54)	1.3 (1.0 – 1.7)	1.1 (0.8–1.5)
	< 16 years	168 (32)	357 (23)	1.9 (1.4 – 2.6)	1.6 (1.2–2.2)
Coloured women^+^		(N = 391)	(N = 1155)		
	≥ 20	60 (15)	265 (23)	[1.0]***	[1.0]***
	16–19 years	200 (51)	616 (53)	1.4 (1.0 – 2.0)	1.2 (0.9–1.7)
	< 16 years	127 (33)	268 (23)	2.1 (1.5 – 3.0)	1.7 (1.2–2.4)
Black women^++^		(N = 133)	(N = 386)		
	≥ 20	16 (12)	40 (10)	[1.0] ***	[1.0]***
	16–19 years	66 (50)	222 (58)	0.7 (0.4 – 1.4)	0.9 (0.5–1.4)
	< 16 years	41 (31)	89 (23)	1.2 (0.6 – 2.3)	1.3 (0.7–2.4)

* Adjusted for number of sexual partners controlled for age, race, marital status, hormonal contraceptive use, years formal education, residence, ever smoked, ever alcohol consumption and ever having had a pap smear

** 14 & 41 women with missing or unknown values for cases and controls respectively are not shown

*** Reference category

^+&++ ^4 & 6 women with missing or unknown values for cases and controls and 10 & 35 women with missing or unknown values for cases and controls respectively are not shown

**Table 5:** Cervical cancer risk in relation to the number of lifetime partners

	Number of life time sexual partners	Cases (n = 524)	Controls (n = 1541)	Crude OR CI (95%)	Adjusted OR* CI (95%)
		N (%)	N (%)		
All women**					
	1	106 (20)	417 (27)	[1.0]***	[1.0]***
	2	157 (30)	521 (34)	1.2 (0.9–1.6)	1.1 (0.8–1.5)
	3	123 (23)	310 (20)	1.6 (1.2–2.1)	1.4 (1.1–1.9)
	≥ 4	133 (25)	285 (18)	1.8 (1.4–2.5)	1.7 (1.2–2.2)
Coloured women^+^		(N = 391)	(N = 1155)		
	1	94 (24)	370 (32)	[1.0]***	[1.0]***
	2	124 (32)	427 (37)	1.1 (0.8–1.5)	1.1 (0.8–1.5)
	3	92 (24)	213 (18)	1.7 (1.2–2.4)	1.5 (1.1 – 2.2)
	≥ 4	80 (20)	142 (12)	2.2 (1.6–3.2)	2.0 (1.4–2.9)
Black women^++^		(N = 133)	(N = 386)		
	1	12 (9)	47 (12)	[1.0***	[1.0]***
	2	33 (25)	94 (24)	1.4 (0.7–2.9)	1.2 (0.6–2.3)
	3	31 (23)	97 (25)	1.3 (0.6–2.7)	1.0 (0.5–2.1)
	≥ 4	53 (40)	143 (37)	1.5 (0.7–2.9)	1.2 (0.6–2.3)

* Adjusted for age of sexual debut controlled for age, race, marital status, use of hormonal contraceptives, years of formal education, area of residence, ever having smoked, ever having drunk alcohol and ever having had a Pap smear

** 5 & 8 women with missing or unknown values for cases and controls respectively are not shown

*** Reference category

^+ and ++ ^1 & 3 women with missing or unknown values for cases and 4 & 5 women with missing or unknown values for cases and controls respectively are not shown

## Results

### Determinants of sexual activity

The median age of sexual debut was 17 years and the median number of lifetime sexual partners was 2 for cases and controls. As can be seen in Table 1 showing a comparison of socio-demographic background of cases and controls, women in both groups had similar characteristics: the mean age was 45 and 44 years respectively; urban residence 58% and 54% and having more 5 or more years of schooling, 76% and 72% respectively; the proportion of married women was 46% and 51% respectively and black and coloured women both 25% and 75% respectively.

Distributions of age of sexual debut and number of sexual partners were similar across major diagnostic categories (trauma, acute infection, other conditions) among controls.

Both tables 2 and 3 show socio-economic factors associated with earlier sexual debut and increased number of partners respectively. These are restricted to controls.

#### Age at sexual debut

Twenty-three percent of the controls had sex < 16 years. The chi square test for trend, an indicator of changes over time, was 1.33, p = 0.25, showing no evidence of a trend of younger sexual debut over time. Table 2 describes socio-demographic factors associated with earlier age at first intercourse and gives crude and adjusted prevalence ratios (APR) for age at first sexual intercourse < 16 years relative to first sex at age ≥ 17 among the controls.

The strongest risk association was for < 4 years of education, (APR = 4.1, 95% CI 2.6–6.6) relative to ≥ 10 years. Other factors significantly associated with age of sexual debut < 16 years, were ≥ 4 sexual partners relative to 1 (APR 1.9, 95% CI 1.3–2.7) and current alcohol use relative to nonuse (APR = 1.4, 95% CI 1.1–1.8); and Age, marital status, race, residence, ever-use of hormonal contraceptives, ever had a Pap smear, smoking status and having current high risk HPV were not significantly associated with early sexual debut. As there was very little reported condom use, few women had knowledge of whether reproductive infections they had had were STI's or not and few women knew whether their partners had had an STI, this had little impact on the analysis.

#### Number of sexual partners

Thirty-nine percent of controls had had at least three sexual partners. Table 3 describes socio-demographic factors associated with an increased number of sexual partners, ≥ 3, relative to 1 partner for among the controls.

Having had ≥ 3 partners was strongly associated with commencement of sexual debut at a younger age (< 16: APR= 2.4, 95% CI 1.8–3.4; 16–19 years: APR= 2.1, 95% CI 1.6–2.9) and being black, (APR = 1.9, 95% CI 1.5–2.3). Other significantly associated factors included being single (APR, 1.3), higher educational levels (APR's for both 5–9 years and ≥ 10 years, 1.3) and current or past alcohol use (APR's 1.6 and 1.7 respectively). Having had ≥ 3 sexual partners was not significantly associated with age, residence, ever-use of hormonal contraceptives, smoking status, having had a Pap smear and having current high risk HPV.

### Sexual activity and cervical cancer risk

Table 4 shows cervical cancer risk, as crude and adjusted odds ratios, in relation to the age at first sexual intercourse.

Relative to those who commenced sexual intercourse at age ≥ 20 years, the multivariate adjusted odds ratio for age at commencement at age < 16 years was 1.6 (95% CI, 1.2–2.2) (p = 0.0006); among coloured and black women the estimates were 1.7 (95 % CI, 1.2–2.4) (p = 0.002) and 1.3 (95% CI 0.7–2.4) respectively.

Table 5 shows cervical cancer risk, as crude and adjusted odds ratios, in relation to the number of lifetime partners.

The multivariate adjusted OR increased with increasing number of sexual partners, to 1.7 (95% CI, 1.2 – 2.2) (p = 0.0001) for ≥ 4 lifetime sexual partners relative to 1. The adjusted OR for cervical cancer in women who had ≥ 4 lifetime sexual partners was 2.0 (95% CI 1.4–2.9) (p < 0.0001) among coloured women and 1.2 (95% CI, 0.6–2.3) among black women. When high risk HPV positive controls only were included (16% of the women), the results were unchanged.

## Discussion

### Determinants of sexual activity

A dearth of information exists on adult women's sexual activity in developing countries. In South Africa cancer of the cervix is a major public health problem and there are high sustained prevalence levels of the oncogenic types of HPV associated with cervical cancer among older women in the country [6]. Information on sexual activity and its determinants are therefore important.

In this study of South African black and coloured women, as for other recent youth studies, [9,10] the median age of sexual debut was 17 for cases and the controls. In keeping with recent international data, our results show no trend of younger sexual debut over time [8]. In addition, age of sexual debut is similar to that shown in a study bringing together international data, including from developed countries [8].

Mostly strongly associated with early sexual debut were lower education, greater number of lifetime sexual partners and alcohol use. Women with low educational levels may have poorer knowledge and a less control over reproductive and sexual decision-making [11]. While it may seem paradoxical that an early sexual debut is associated with lower educational levels while having a greater number of partners is associated with the inverse, this is not necessarily the case. Higher education may increase a woman's financial autonomy, allowing her to have greater independence in choice in intimate relationships and hence increase her number of life-time partners. Early pregnancy limits women's educational prospects and may in turn limit employment opportunities and create lower perceptions of life chances and decreased incentive to delay childbearing [11,12]. Urban and rural South African studies have found education, employment and earning opportunities significantly reduced risk-taking behavior [13,14]. Less educated young women may face greater constraints engaging in safer sex [15]. Alcohol use could indicate riskier life styles. While smoking and cervical cancer risk has been associated in some studies, [16,17] we found no association, but the median number of cigarettes per day reported by smokers in this study was low.

Factors most strongly associated with having more sexual partners were being single, black, more educated, use of alcohol, and young age at first sex. Being single creates more opportunity for having more partners, as does younger sexual debut. Higher education may increase women's financial autonomy, affecting number of sexual partners.

Black women's sexual behaviour may be influence by historical socio-political inequality. Apartheid migratory labour practices and the pass laws that controlled the movement of Black South Africans, severely restricting movement outside of rural areas, created separation of black households, placing women at risk for multiple sexual partners [18,19]. Women's economic constraints also led to a greater prevalence of women in urban areas engaging in transactional sex [18]. Current infection with high risk HPV was not found to be significantly associated with sexual risk behaviour. While it would be expected that the two variables studies would show an association, it was possible in this study only to accertain current and not life-time exposure to HPV infection.

### Sexual activity and cervical cancer risk

In agreement with previous studies conducted internationally early age of sexual debut and a large numbers of sexual partners were associated with increased cervical cancer risk in the present study [21-23]. The ORs were larger for coloured women than for black women. We may have expected a similar association between sexual activity and cervical risk for both groups. However, the number of black women was limited, confidence intervals were wide, and odds ratios in the two groups were compatible with a uniform value. An alternative explanation may be confounding by the sexual activity of the male partners. Because of migratory labour, black women's male partners may have more partners than coloured women's male partners, increasing the risk of transmission of HPV infection, and of cervical cancer. We had no data on male partners' sexual activity. Studies examining male partners' their sexual behaviour show these to have a greater impact on cervical cancer risk than the women's own behaviour. [24,25]. Data collected on contraceptive history included condom use. However this was very low among this population of women. Data was also collected on whether women and their partners had ever had a sexually transmitted infection, with no marked effects evident, possibly due to the high life time prevalence of reproductive infections among women. The information on partners' STI's was unlikely to be reliable. The study could only obtain endocervical scrapings for HPV from controls and not cases, due to the latter presenting in South Africa with advanced disease [7]. Hence any attenuation of the sexual behavior through inclusion of HPV status in the multivariate models could not be verified.

Controls in this study were age matched cases of cervical cancer, a disease more common in older women. Hence the study is limited in making inferences about younger women since only a small percent of the study population (6%) was under the age of 30 years.

It is important to consider whether the present findings are due to bias. Refusal rates were low, reducing the possibility of selection bias. Additionally, the distributions of sexual activity were similar across the diagnostic categories in the controls, suggesting that no bias in the selection of controls. Regarding detection bias, most cases of women with cervical cancer would be treated at hospitals monitored by the study, because the study was confined to women with invasive cancer (Stage 1B – IV) for whom diagnosis and hospital admission was inevitable. To guard against information bias, standardized interviews were administered to cases and controls in similar settings. However, questions about sexual activity are intrinsically sensitive and sexual activity has many determinants such as culture, religion and socio-economic status that may limit precision. Thus the potential for information bias is acknowledged. Confounding is also a possibility. While we controlled for major risk factors, we could not control and rule out confounding for sexual activity of the male partners.

## Conclusion

This study provides valuable information on patterns and determinants of sexual activity in a population of South African adult women. This is relevant in understanding the high levels of cervical cancer among women in South Africa and other developing countries and more broadly in examining STI acquisition, including HIV that has high prevalence levels among older South African women in their reproductive years [26]. In this study low socio-economic status, alcohol intake, single marital status and being black, most likely related to historical socio-political inequality, appear to be determinants of increased sexual activity. As expected, such activity was also associated with an increased risk of cervical cancer, confirming the findings of studies conducted in other countries. They suggest that initiatives to encourage later commencement of sex, and limit the number of sexual partners would have a favourable impact on risk of cancer of the cervix and other STI's, including HIV. The findings underscore the importance of calls made elsewhere for public health interventions for sexual risk reduction to pay greater attention to the social context in which sexual activity occurs [8].

## Competing interests

The author(s) declare that they have no competing interests.

## Authors' contributions

All authors have been involved either in the conception, design of the study and/or in the analysis and interpretation of data. SS, LR, MH were involved in the conception and design of the study. DC, MH, AW, LD & GD were involved in the acquisition of data. HC, JK & IS performed statistical analysis. All authors were involved in analysis and interpretation of data. The first author drafted the manuscript all authors have been involved in critically revising the article for important intellectual content and have given final approval to the version to be published.

## Pre-publication history

The pre-publication history for this paper can be accessed here:


